# The psychological burden of COVID-19 on the desire for parenthood in minoritized sexual identities: a study on depressive symptoms and family planning in Germany

**DOI:** 10.1186/s12889-023-15127-7

**Published:** 2023-02-02

**Authors:** Falk Batz, Eva Lermer, Sonia Lech, Grace O’Malley, Alaleh Zati zehni, Davina Zenz-Spitzweg, Sven Mahner, Joachim Behr, Christian J. Thaler, Pichit Buspavanich

**Affiliations:** 1 Department of Obstetrics and Gynecology, Center for Gynecological Endocrinology and Reproductive Medicine, University Hospital, LMU Munich, Munich, Germany; 2 Center for Leadership and People Management, LMU Munich, Munich, Germany; 3 Department of Business Psychology, Augsburg University of Applied Sciences, Augsburg, Germany; 4 Institute of Medical Sociology and Rehabilitation Science, Charité – Universitätsmedizin Berlin, corporate member of Freie Universität Berlin and Humboldt- Universität zu Berlin, Berlin, Germany; 5 Department of Psychiatry and Psychotherapy, Charité – Universitätsmedizin Berlin, corporate member of Freie Universität Berlin and Humboldt-Universität zu Berlin, Berlin, Germany; 6 Department of Paediatric Oncology/Haematology, Charité – Universitätsmedizin Berlin, corporate member of Freie Universität Berlin and Humboldt-Universität zu Berlin, Berlin, Germany; 7 Department of Psychiatry, Psychotherapy and Psychosomatics, Brandenburg Medical School Theodor Fontane, Fehrbelliner Str. 38, 16816 Neuruppin, Germany; 8 Faculty of Health Sciences Brandenburg, Joint Faculty of the University of Potsdam, Brandenburg University of Technology Cottbus-Senftenberg and Brandenburg Medical School, Potsdam, Germany; 9 Research Unit Gender in Medicine, Department of Psychiatry and Psychotherapy, Institute of Sexology and Sexual Medicine, Charité – Universitätsmedizin Berlin, corporate member of Freie Universität Berlin and Humboldt-Universität zu Berlin, Berlin, Germany; 10 Applied Business and Media Psychology, Ansbach University of Applied Sciences, Ansbach, Germany

**Keywords:** COVID − 19, Minoritized sexual identities, Minoritized gender identities, Motives for parenthood, Desire for parenthood

## Abstract

**Background:**

The COVID-19 pandemic continues to spread across the globe and is associated with significant clinical and humanitarian burden. The desire for parenthood has been described to be positively correlated with psychological well-being: An unfulfilled wish for parenthood is associated with impaired mental health, and the wish for parenthood is a predictor for the development of depressive symptoms. While higher rates of anxiety and depression have been reported in individuals with minoritized sexual identities (compared to heterosexual individuals) during the COVID-19 pandemic, the specific impact of the pandemic and its social restriction measures on this population is poorly understood.

**Methods:**

From April to July 2020, we conducted an anonymous cross-sectional survey online among N = 2463 adults living in Germany. We screened for depressive symptoms (Patient Health Questionnaire-4; PHQ-4) and assessed individuals’ desire for parenthood during the pandemic, and motives for or against the desire for parenthood (Leipzig questionnaire on motives for having a child, Version 20; LKM-20), with the aim of identifying differences between individuals with minoritized sexual identities and heterosexual individuals.

**Results:**

Compared to heterosexual individuals (n = 1304), individuals with minoritized sexual identities (n = 831) indicated higher levels of depressive symptoms. In our study sample the majority of all participants (81.9%) reported no change in the desire for parenthood since the COVID-19 pandemic.

**Conclusion:**

The findings underline the unmet need for social, psychological and medical support in regard to family-planning and the desire for parenthood during a pandemic. Furthermore, future research should explore COVID-19-related psychological consequences on individuals’ desire for parenthood and building a family.

## Background

The severe acute respiratory syndrome coronavirus type 2 (SARS-CoV-2) is the novel coronavirus disease (COVID-19) which, at the time of writing in December 2022, has caused approximately 6.6 million deaths worldwide. Besides its physical consequences, it has an immense psychological effect on citizens and livelihoods across the entire globe [1]. On 22nd March 2020 the first measures of social isolation including home confinement were declared by the German federal states. Individuals who sexually-identify as other than exclusively heterosexual (sexual minorities) constitute an at-risk group both in terms of their physical and mental health [2, 3]. Thus, the impact of the COVID-19 pandemic and the associated social isolation measures (including at-home confinement) on sexual minorities is especially devastating [4–6]: Individuals with minoritized sexual identities are reported to show significantly lower well-being and significantly higher rates of emotional disorders such as depressive symptoms in comparison to heterosexual individuals since the COVID-19 pandemic [7–10]. While stable and supportive relationships (e.g., family or partners) have been highlighted as protective against psychological difficulties [11, 12], access to one’s social supports and relationships have often been curtailed by confinement measures set out by legislation. In turn, individual vulnerability to psychological distress and the onset or exacerbation of emotional disorders such as depression increases and can have a further negative impact on couple stability, reproductive health and the desire for parenthood [13, 14]. To this end, it comes as no surprise that social-distancing measures and personal isolation have been shown to greatly impact physical intimacy and reproductive health since the onset of COVID-19 [15, 16]. Intimate and familial relationships have been strained by both imposed, prolonged periods of separation, or indeed the opposite; namely a dramatic increase in time spent together in a confined space. Such stark changes may have a positive or negative impact on interpersonal relations by influencing the vitality of relationships, reproductive health and the wish to become a parent [17, 18].

The desire for parenthood has been described to be positively correlated with psychological well-being: An unfulfilled wish for parenthood is associated with impaired mental health and the wish for parenthood is a predictor for developing depressive symptoms [19, 20]. The direction of the association between depression and unfulfilled parenthood needs further investigation. Initial data on the impact of the COVID-19 pandemic on the desire for parenthood show a significant decrease of birth rates after the outbreak of the COVID-19 pandemic mainly due to worries of future economic difficulties and potential consequences [13, 21, 22], which is consistent with previous literature indicating that lower income negatively affects the desire for parenthood [23].

Despite the fact that marriage and adoption rights have been open to same-sex couples since 2017 in Germany, individuals with minoritized sexual identities continue to endure additional obstacles relative to heterosexual parents in their journey to parenthood, including limited national legal possibilities [24].

To date, research on parenthood and family planning has often focused on heterosexual individuals. However, existing literature on parenthood desires among minoritized sexual identities has shown mixed results: While motives for and against having children do not differ between heterosexuals and individuals with minoritized sexual and gender identities [25–27], individuals of minoritized sexual identities tend to think longer about the motives and meaning of their wish to become parents [26, 28]. Further, data on same-sex couples indicate no or little differences in motives for or against becoming a parent compared to heterosexual individuals [27, 29]. However, recent empirical work repeatedly reported lower desires for parenthood in minoritized sexual identities compared to heterosexuals [30, 31]. To sum up, the association between parenthood desires and sexual identity as well as factors impacting this association remain unclear, especially during the COVID-19 pandemic.

### Aim of the present study

The present study aims to examine the impact of the COVID-19 pandemic and the precautionary social restriction measures in Germany on decisions regarding family- planning among cis-heterosexuals and cis-individuals with minoritized sexual identities.

Based on previous literature, the following research questions and hypotheses are proposed: Individual’s overall levels of depressive symptoms are higher among individuals with minoritized sexual identities compared to a cis-heterosexual population in Germany during the COVID-19 pandemic (Hypothesis 1). Secondly, we want to explore whether individuals’ current desire for parenthood has decreased during the COVID-19 pandemic as compared to beforehand (Research Question 1). Third, we proposed that the desire for parenthood in individuals with minoritized sexual identities compared to a cis-heterosexual population in Germany has decreased since the COVID-19 pandemic (Hypothesis 2). Further, we aimed to explore the motives for and against the desire for parenthood in heterosexual cis-individuals compared to cis-individuals with minoritized sexual identities during the COVID-19 pandemic (Research Question 2). Lastly, we aimed to examine the influence of sexual orientation, gender, age, residential environment, relationship status, parenthood, current score of depressive symptoms and motives for or against having children on the desire for parenthood (Research Question 3).

## Methods

### Setting, study design and sample

An anonymous, cross-sectional open-access survey was generated online and made available nationwide in Germany using SoSci Survey (Version 3.2.23). The survey was administered in German language and shared via online invitations. A link with access to the survey was distributed within social communication networks, including in threads on FacebookTM, InstagramTM, TwitterTM and WhatsAppTM. Additionally, the survey was distributed across several websites of sexual and gender minority organizations and magazines. Some participants promoted the survey within their own local social media networks (snowball sampling). Prior to data collection, all participants provided full consent to participate online after reviewing the online information material. Study participation was anonymous, voluntary, and uncompensated. The survey was registered by the Ethics Review Committee of the Faculty of Medicine, LMU Munich (registration number: 20–344 KB) and conducted with accordance to the Declaration of Helsinki. Further, we adopted recommendations set out in the *Sex And Gender Equity in Research* (SAGER) guidelines [32].

### Measures

*Gender identity and sexual orientation*: Gender identity and sexual orientation were assessed with the item “*In your opinion, which of the following categories most apply to you?*”, for which the following response categories were provided: *heterosexual, homosexual, bisexual, asexual, female, male, cis* (“*I identify with the gender assigned at birth*”), trans* (“*I do not identify with the gender assigned at birth*”) and *others*. Note that we are aware of the pathologizing nature of the term “homosexual”. In Germany, the term “*homosexuell*” is still widely used; for reasons of transparency, we report the direct translation as it appeared in the survey question. Multiple answers were possible. For the purpose of the analysis, we divided all cis-participants into 8 groups according to their self-assigned gender identity and sexual orientation: (I) heterosexual women, (II) heterosexual men, (III) lesbian women, (IV) gay men, (V) bisexual women, (VI) bisexual men, (VII) asexual women, (VIII) asexual men. The two analytical groups of this study are: *individuals with minoritized sexual identities* (lesbian women, gay men, bisexual women, bisexual men, asexual women and asexual men) and *heterosexual individuals* (heterosexual women and heterosexual men). Due to the complexity of gender and sexual identity and the low number of trans* participants we did not include this group in the current study.

Further, the respondents were asked if they had children (*yes, no*) and if they have a desire for parenthood (*yes, no*). To examine the impact of the COVID-19 pandemic on the desire for parenthood, participants were asked if the desire for parenthood has [1] *increased*, [2] *decreased* or [3] *not changed*.

#### Depressive symptoms

We used the 4-item Patient Health Questionnaire-4 (PHQ-4), a standardized, brief, self-report questionnaire to measure current depressive symptoms [35]. The four items assess the subjective psychological level of depressive symptoms over a 14-day period (“Feeling nervous, anxious, or on edge”; “Not being able to stop or control worrying”, “Feeling down, depressed, or hopeless”; “Little interest or pleasure in doing things”). Responses are scored from (0) “Not at all”, [1] “Several days”, [2] “More than half the days” or [3] “Nearly every day”. The total raw score ranges from 0 to 12, whereby higher values indicate higher levels of depressive symptoms (with a total raw score ≥ 6 indicating moderate to severe depressive symptoms) [36]. Löwe et al. validated the PHQ-4 in the general population with a sample that closely matches the sociodemographic characteristics of Germany as well as those in the United States. A cut-off value of 6 is recommended for the presence of clinically-relevant depression and/or anxiety, as it corresponds to the 96th percentile value [33]. The PHQ-4 total score measures symptom load as well as the grade of functional impairment and possible disability in everyday life due to depressive symptoms [36]. An elevated score in PHQ-4 does not serve as a diagnostic tool, but rather as a screener which indicates whether further diagnostic assessment is necessary [34]. In the present study, the scale showed very good internal consistency (Cronbach’s alpha = 0.848).

*Motives for and against having children*: The motives for and against parenthood were assessed by means of the Leipzig questionnaire on motives for having a child, Version 20 (LKM-20) [35]. The LKM-20 is a 20-item questionnaire investigating motives in favor of and against having children. Participants were asked to what extent a certain motive influences their personal decisions for or against having children. Their attitudes toward a given motive for becoming a parent were assessed using the following thresholds: [1] *Does not influence me at all*, [2] *Does moderately influence me*, [3] *Does partially influence me*, [4] *Does reasonably influence me*, [5] *Does strongly influence me*. The items form four scales, characterized as follows: Scale 1. The desire for emotional stability and life meaning (e. g. “*A child gives me the feeling of having a real home’’*; Cronbach’s alpha = 0.914); Scale 2. Fear of personal constraints Cronbach’s alpha = 0.756); Scale 3. The desire for social recognition (Cronbach’s alpha = 0.921); Scale 4. Fear of financial constraints (Cronbach’s alpha = 0.815). The LKM-20 has been proven to be a reliable, valid, and economical instrument of practical value with data obtained from a representative sample of the whole German population between the ages of 16 and 45 years [35].

### Participants

Between April and July 2020, a total of N = 2463 participants took part in the online survey. To maximize participation, the inclusion criteria were held broad and limited to [1] a minimum age of 18 years and [2] knowledge of German language. Due to missing values, n = 328 participants had to be excluded from the analysis. In total, this resulted in N = 2135 participants eligible for inclusion in the analysis. Items measuring socio-demographics and other covariates were treated as categorical variables. Most participants were 35 years or younger (n = 1682, 78.9%). In terms of employment status, most participants across all three groups n = 1234 (57.3%) were currently working and a total of n = 761 (35.7%) participants were students. The majority of participants n = 1647 (77.3%) lived in urban cities of 20,000 inhabitants or more. 71.7% of heterosexual participants (n = 935) and 66.1% of individuals of minoritized sexual identity (n = 549) were currently in a relationship. Only n = 8 participants reported a current COVID-19 infection and n = 13 reported a previous COVID-19 infection. For more details, see descriptive statistics in Table 1.

### Statistical analyses

This study focuses on the impact of the COVID-19 precautionary measures on family planning and motives for or against becoming a parent in individuals with minoritized sexual identities compared to heterosexual individuals. First, descriptive statistics were calculated for all subgroups (I) heterosexual women, (II) heterosexual men, (III) lesbian women, (IV) gay men, (V) bisexual women, (VI) bisexual men, (VII) asexual women, (VIII) asexual men and the variables of interest (Table 1). To test Hypothesis 1, we conducted a two-sample t-test for the comparison of the groups cis-heterosexual versus not cis-heterosexual as well as an ANOVA with posthoc tests (LSD) for more differentiated insights. Descriptive data were presented in Table 1 to report potential changes in the desire for parenthood since the COVID-19 pandemic (Research Question 1). To examine whether cis-individuals with minoritized sexual identities have less desire for parenthood compared to cis-heterosexuals during the pandemic a chi-square test has been performed (Hypothesis 2). Further, to compare motives for and against having a child in individuals with minoritized sexual identities and heterosexual individuals during the COVID-19 pandemic, two-sample t-tests were performed (Research Question 2). Finally, we examined the associations between the subgroups and the current motives for and against having a child by conducting a multivariate logistic regression model, whereby the desire for parenthood represented the dichotomous, dependent variable (Research Question 3). We used dichotomous variables for heterosexual individuals and individuals with minoritized sexual identities whereby heterosexual served as the reference group. We included the following covariates in the model: *gender* (women versus men), *sexual orientation* (heterosexual versus individuals with minoritized sexual identities), *age* (18–25 years, 26–35 years versus 36 years and above), *residential environment* (urban cities versus rural communities under 20,000 inhabitants), *relationship status* (single versus in a relationship), *parenthood* (yes versus no), *depressive symptoms* (PHQ-4 total raw score), *motives for and against the desire for parenthood* (sum-scores of each LKM-20 scale). Basic assumptions for logistic regression including independence of errors, linearity in the logit for continuous variables, absence of multicollinearity, and lack of strongly influential outliers were tested and suitable for this analysis. The Kolmogorov-Smirnov test was used to verify the normal distribution of the sample for metric variables. All tests of significance were based on a *p* < 0.05 level and a confidence interval of 95%. Statistical analysis was performed with SPSS Version 26.

## Results

### Depressive symptoms

To test Hypothesis 1, a two-sample t-test revealed that the cis-heterosexual group had a significantly lower level of depression (M = 3.43, SD = 2.61) compared to participants who assigned themselves to a sexual minority (M = 4.08, SD = 2.78):

t(2078) = -5.433 p = < 0.001, d = 0.242.

In terms of the prevalence of moderate to severe depressive symptoms, bisexual participants reported the highest levels (n = 126, 40.7%), followed by asexual participants (n = 11, 33.3%). The lowest level of depressive symptoms was reported by heterosexual individuals (n = 227, 20.1%) and lesbian or gay individuals (n = 74, 18.5%). ANOVA results showed a significant group effect indicating significant differences in depressive symptoms between the study groups: F(7, 2072) = 12.94,

*p* < 0.001. LSD posthoc tests revealed that bisexual women (*p* < 0.001) and asexual women (*p* < 0.001) had significantly lower scores than heterosexual women and bisexual men (*p* < 0.001) had significantly lower scores than heterosexual men (heterosexual women: *M* = 3.39, *SD* = 0.165; heterosexual men: *M* = 3.53, *SD* = 0.176; bisexual women: *M* = 4.64, *SD* = 0.187; bisexual men: *M* = 5.33, *SD* = 0.338; asexual women: *M* = 5.27, *SD* = 0.497).

**Table 1:** Descriptive statistics of the sample. N = 2135; n (%), mean [standard deviation].

	Heterosexual individuals		Individuals with minoritized sexual identities					
	**Heterosexual women**	**Heterosexual men**	**Lesbian women**	**Gay men**	**Bisexual women**	**Bisexual men**	**Asexual women**	**Asexual men**
	1004 (47.0)	300 (14.1)	353 (16.5)	108 (5.1)	254 (11.9)	80 (3.7)	29 (1.4)	7 (0.3)
**Age**								
18–25 years	389 (38.8)	123 (41.0)	90 (25.5)	18 (16.7)	95 (37.4)	25 (31.3)	14 (48.3)	3 (42.9)
26–35 years	476 (47.5)	118 (39.9)	141 (39.9)	50 (46.3)	102 (40.2)	24 (30.0)	12 (41.4)	2 (28.6)
36–45 years	116 (11.6)	35 (11.7)	93 (26.3)	19 (17.6)	46 (18.1)	26 (32.5)	0 (0.0)	2 (28.6)
46 years and above	21 (2.1)	24 (8.0)	29 (8.2)	21 (19.4)	11 (4.3)	5 (6.3)	3 (10.3)	0 (0.0)
**Relationship status**								
Single	267 (26.6)	102 (34.0)	81 (22.9)	33 (30.6)	100 (39.4)	43 (53.8)	19 (65.5)	6 (85.7)
In a relationship	737 (73.4)	198 (66.0)	272 (77.1)	75 (69.4)	154 (60.6)	37 (46.3)	10 (34.5)	1 (14.3)
**Residential environment**								
Urban metropolis^a^	674 (67.3)	184 (61.3)	178 (50.6)	67 (62.0)	153 (60.5)	58 (72.5)	18 (62.1)	6 (85.7)
Medium-sized town^b^	131 (13.1)	46 (15.3)	73 (20.7)	12 (11.1)	34 (13.4)	7 (8.8)	6 (20.7)	0 (0.0)
Small town^c^	107 (10.7)	40 (13.3)	44 (12.5)	23 (21.3)	31 (12.3)	10 (12.5)	3(10.3)	0 (0.0)
Rural community^d^	90 (9.0)	30 (10.0)	57 (16.2)	6 (5.6)	35 (13.8)	5 (6.3)	2 (6.9)	1 (14.3)
**Employment status**								
Self-employed	53 (5.3)	11 (3.7)	23 (6.5)	6 (5.6)	14 (5.5)	4 (5.0)	5 (17.2)	0 (0.0)
Employed	464 (46.3)	135 (45.2)	244 (69.3)	80 (74.8)	135 (53.1)	49 (61.3)	8 (27.6)	3 (42.9)
Student	425 (42.4)	136 (45.5)	59 (16.8)	18 (16.8)	82 (32.3)	23 (28.7)	14 (48.3)	4 (57.1)
Not employed	60 (6.0)	17 (5.7)	26 (7.4)	3 (2.8)	23 (9.1)	4 (5.0)	2 (6.9)	0 (0.0)
**COVID-19 status**								
Infected, symptomatic	0 (0.0)	2 (0.7)	0 (0.0)	1 (1.0)	0 (0.0)	0 (0.0)	0 (0.0)	0 (0.0)
Infected, asymptomic	2 (0.2)	0 (0.0)	1 (0.3)	1 (1.0)	0 (0.0)	1 (1.3)	0 (0.0)	0 (0.0)
Previously infected	7 (0.7)	2 (0.7)	2 (0.6)	0 (0.0)	0 (0.0)	1 (1.3)	0 (0.0)	1 (16.7)
Not infected/not tested	983 (99.1)	294 (98.7)	337 (99.1)	101 (98.1)	250 (100)	74 (97.4)	29 (100)	5 (83.3)
**Depressive symptoms**								
PHQ-4^e^ total raw score	3.39 [2.59]	3.53 [2.65]	3.57 [2.60]	3.24 [2.76]	4.64 [2.74]	5.34 [2.93]	5.28 [2.92]	2.5 [2.07]
**Clinical relevant depression** ^f^								
No	685 (79.6)	209 (80.4)	255 (81.7)	71 (80.1)	156 (65.5)	27 (38.1)	18 (62.1)	4 (100)
Yes	176 (20.4)	51 (19.6)	57 (18.3)	17 (19.9)	82 (34.5)	44 (71.2)	11 (37.9)	0 (0.0)
**Parenthood**								
No	823 (87.6)	223 (82.0)	250 (77.2)	87 (87.0)	202 (84.2)	44 (62.0)	24 (85.7)	5 (100)
Yes	117 (12.4)	49 (18.0)	74 (22.8)	13 (13.0)	38 (15.8)	27 (38.0)	4 (14.3)	0 (0.0)
**Desire for parenthood**								
No	100 (10.7)	35 (12.9)	90 (27.8)	27 (27.0)	48 (20.0)	5 (7.0)	7 (25.0)	1 (20.0)
Yes	837 (89.3)	235 (87.1)	234 (72.2)	73 (73.0)	192 (80.0)	66 (93.0)	21 (75.0)	4 (80.0)
**Desire for parenthood since the COVID-19**^**g**^ ** pandemic**								
Increased	93 (10.0)	29 (10.7)	20 (6.2)	17 (17.5)	19 (8.0)	9 (12.7)	1 (3.7)	4 (80.0)
Decreased	100 (10.7)	12 (4.4)	26 (8.1)	7 (7.2)	12 (5.1)	4 (5.6)	2 (7.4)	0 (0.0)
Did not change	740 (79.3)	230 (84.9)	275 (85.7)	73 (75.3)	206 (86.9)	58 (81.7)	24 (88.9)	1 (20.0)

Notes: a. 100.000 or more inhabitants; b. 20.000 to 100.000 inhabitants; c. 5.000 to 20.000 inhabitants; d. up to 5.000 inhabitants; e. f. Patient Health Questionnaire 4 items (PHQ-4); f. A total raw score PHQ-4 ≥ 6 indicates moderate to severe depressive symptoms; g. Item “Desire for parenthood since the COVID-19”: Participants were asked if the desire for parenthood has (1) increased, (2) decreased or (3) not changed.

### Parental status and family planning

Most respondents (83.7%) reported having no children (women 87.6%, men 82%, individuals with minoritized sexual identities 79.7%). To explore whether the desire for parenthood had changed, participants were asked if they have a desire for parenthood and if their desire for parenthood had increased, decreased or not changed since the pandemic (Research Question 1). A total of 1667 participants (84.2%) reported a desire for parenthood. As shown in Table 1, more heterosexuals (n = 1072, 88.8%) stated that they had decided to have children compared to individuals with minoritized sexual identities (n = 590, 76.8%). Looking at the whole sample, 163 respondents (8.3%) described a decrease and 192 (9.8%) reported an increase in the desire for parenthood since the COVID-19 pandemic. No change in the desire for parenthood since the COVID-19 pandemic was reported for participants overall (n = 1607, 81.9%).

Further results revealed that more participants from the group cis-heterosexual indicated a desire for parenthood compared to participants from the group of cis individuals with minoritized sexual identities (Hypothesis 2, desire for parenthood: 88.81% cis-heterosexuals (n = 1072), 76.82% cis-individuals with minoritized sexual identities (n = 590); no desire for parenthood: 11.18% cis-heterosexuals (n = 135), 23.17% cis-individuals with minoritized sexual identities; (n = 178; p < 0.001).

**Table 2:** Comparison of motives for and against having a child in individuals with minoritized sexual identities and heterosexual individuals during the COVID-19 pandemic

LKM-20 Subscales scores		*M*	*SD*	*p*	*d*
(1) Desire for emotional stabilization	Individuals with minoritized sexual identities	2.77	1.36	< 0.001	0.183
	Heterosexual individuals	3.01	1.26		
(2) Fear of personal constraints	Individuals with minoritized sexual identities	1.99	0.84	0.133	0.083
	Heterosexual individuals	2.06	0.85		
(3) Desire for social recognition	Individuals with minoritized sexual identities	1.44	0.81	0.048	0.100
	Heterosexual individuals	1.52	0.79		
(4) Fear of financial constraints	Individuals with minoritized sexual identities	2.41	0.98	0.523	0.031
	Heterosexual individuals	2.44	0.97		

Notes: M = mean; SD = standard deviation; p = p-value; d = Cohen’s d

### Motives for and against having children (LKM-20)

Table 2 presents a comparison of motives for and against having a child in individuals with minoritized sexual identities and heterosexual individuals during the COVID-19 pandemic since the COVID-19 pandemic (Research Question 2). For both participant groups, the ranking of motives for and against the desire for parenthood was similar [36]. From all areas of the LKM-20, the motive “Desire for emotional stability and life meaning” (Scale 1) had the greatest influence on the desire for own parenthood [25]. This motive was followed by Scale 4: “Fear of financial constraints”. Scale 2 “Fear of personal constraint” was less significantly endorsed. Subsequently, Scale 3 “Desire for social recognition” showed the least influence on the desire for parenthood. Furthermore, for all scales except *Fear of Financial Constraints*, respondents with minoritized sexual identities scored significantly lower than heterosexual respondents. Finally, to explore the influence of gender, sexual orientation, age, employment status, relationship status, parenthood, depressive symptoms (PHQ-4) and motives for and against becoming parents (LKM-20) in connection with the wish for having children, we performed a binary logistic regression model (See Table 3). Results revealed a significant negative association between the desire for parenthood and minoritized sexual identities compared to heterosexual individuals. Having one or more children, younger age and living in an urban city were significantly positively associated with the desire to become a parent. In regards to motives for wishing to become a parent, *Desire for emotional stability and life meaning* was significantly positively associated with the desire for parenthood, whereas *Fear of personal constraints* and *Desire for social recognition* were significantly negatively correlated with the wish for parenthood in this model. Furthermore, lower depressive symptoms (PHQ-4) were significantly associated with the desire for parenthood. All other covariates had no significant effect.

**Table 3:** Multivariate logistic regression model with desire for parenthood as dependent variable

	Regression coefficient	*Standard Error*	*p*	*Odds* *ratio*	95% Confidence Interval Lower Upper	
Sexual orientation^a^	-1.130	0.203	< 0.001	0.323	0.217	0.481
Gender^b^	0.079	0.241	0.744	1.082	0.674	1.737
Age group			0.001			
18–25 years	1.157	0.316	< 0.001	3.181	1.712	5.911
26–35 years	0.731	0.303	0.016	2.078	1.147	3.765
Over 35 years (reference)	-	-	-	-	-	-
Relationship status^c^	0.114	0.212	0.591	1.121	0.740	1.697
Residential environment^d^	-0.438	0.210	0.037	0.645	0.427	0.974
Parenthood^e^	2.256	0.528	< 0.001	9.543	3.393	26.838
PHQ-4 total score	-0.106	0.039	0.007	0.900	0.833	0.972
LKM-20 subscale 1	2.357	0.178	< 0.001	10.563	7.451	14.974
LKM-20 subscale 2	-0.622	0.162	< 0.001	0.573	0.391	0.737
LKM-20 subscale 3	-0.751	0.222	< 0.001	0.472	0.306	0.729
LKM-20 subscale 4	0.084	0.144	0.561	1.087	0.820	1.442
Intercept	-1.017	0.456	0.026	0.362		
**Statistics for logistic regression**						
Nagelkerke’s R^2^	0.628					
Standard Error	0.069					

Notes: p = p-value; a. 1 = heterosexual, 2 = individuals with minoritized sexual identities; b. 1 = women, 2 = men; c. 1 = single, 2 = in a relationship; d. 1 = urban cities, 2 = rural communities under 20.000 inhabitants; e. 1 = no children; 2 = one or more children, f. depressive symptoms were measured with the Patient Health Questionnaire 4 items (PHQ-4) total raw score

## Discussion

The present study focused on the impact of the COVID-19 pandemic on mental health, the desire for parenthood, and various motives for and against having a child in individuals with minoritized sexual identities compared to heterosexual individuals.

Firstly, we confirmed previously published data on higher scores of depressive symptoms in individuals with minoritized sexual identities in times of a social crisis such as the COVID-19 pandemic, as compared to heterosexuals [2, 37]. This is in line with the Minority Stress Model whereby individuals belonging to minoritized groups are exposed to unique stress related to their race, gender or sexual orientation leading to mental health issues such as depression or anxiety [38].

In taking a closer look at our subgroups, we found significantly higher levels of depressive symptoms in bisexual and asexual individuals compared to heterosexual, lesbian and gay individuals. This finding is not surprising, given that previous empirical work has suggested asexual and bisexual individuals to be at particular risk of poor mental health among all individuals with minoritized sexual identities [3, 39]. Notably, we did not find a significant difference between lesbian, gay and heterosexual individuals in regard to depressive symptoms in our data. However, existing literature would generally suggest that gay and lesbian individuals report greater mental distress than heterosexual individuals during the COVID-19 pandemic [40]. Thus, this finding should be interpreted cautiously. It is possible that our findings may be explained by selection bias, whereby particularly low-adjusted heterosexual participants self-selected for participation in our study.

Secondly, since literature has indicated that the desire for parenthood positively correlates with psychological well-being, and that the desire for parenthood is negatively associated with depression [19, 20], we expected to find a decreased desire for parenthood due to the increase in depressive symptoms since the COVID-19 pandemic, especially in individuals with minoritized sexual identities. In our sample, most participants reported no change in the desire for parenthood since the COVID-19 pandemic. In accordance with previous studies, individuals with minoritized sexual identities (63.6%) indicated less desire for parenthood compared to heterosexual individuals (76.8%). To compare, the general desire for parenthood was 87.2% in German and 92.3% in Chinese participants in a study sampling 13,498 students [41]. Similar to our results, a study on Czech lesbians sampled between 2013 and 2017 indicated that 15.6% of the participants did not desire parenthood [42]. In a study from the United States, 52.0% of gay men and 41.0% of lesbian women without children reported wanting to become a parent [43]. The lower levels of desire for parenthood in individuals with minoritized sexual identities compared to heterosexuals can be explained by the fact that there are more obstacles to become parents for individuals of sexual minoritized identities: External factors such as legal constraints and social acceptance, and internal factors such as internalized stigmatization, may play a role in individuals’ mental health and cause extra hindrances with which heterosexual couples do not have to contend [44, 45]. Contrary to our findings, a retrospective study from Italy with 1482 heterosexuals living in a stable relationship for at least twelve months documented that 37.3% of respondents abandoned their plan to become a parent since the COVID-19 pandemic. In particular, worries of future economic difficulties and potential consequences have previously been described as reasons for a decrease in the desire for parenthood [13, 21, 23, 46].

Curiously and contrary to the aforementioned results, the birth rate in Germany rose since the pandemic. The German Federal Statistical Office reported the highest birth rates in 20 years in Germany in March 2021. Compared with a year prior, the number of births rose by 10% in March 2021 indicating that the desire for parenthood and the intention to start a family rose despite the COVID-19 pandemic and its confinement measures. On this basis, the COVID-19 pandemic and the associated increase in individuals’ subjectively experienced uncertainty, lower mental well-being and higher rates of depressive symptoms was associated with an increase in birthrates in Germany. While this may seem counter-intuitive, it is in line with previous studies documenting that traumatizing, life-threatening and indeed high-mortality events such as the Gujarat earthquake in India in 2001 and the Hurricane Mitch in Nicaragua in 1998 [47, 48] can in fact result in higher birth rates.

Third, in an exploratory analysis we compared our results to data by Kleinert et al. 2015: The ranking of the motives for parenthood in both heterosexual and minoritized sexual identity individuals did not differ since the COVID-19 pandemic (data not shown in the results section). Further, both subgroups in the present study scored lower in all dimensions as compared to the 2015 data. These findings are in accordance with previous studies where literature shows that motives for and against having children do not differ between sexual or gender identities [25–27]. Consistent with our results, data from 2015 by Kleinert et al. defined that the most important influence on the motives for parenthood was the desire for emotional stabilization [25]. With regard to individuals with minoritized sexual identities, data on same-sex couples indicate no or little differences in motives for or against becoming a parent compared to heterosexual individuals [27, 29]. Yet, individuals of minoritized sexual identities tend to think longer about the motives and meaning of their wish to become parents [26, 28]. One could postulate that COVID-19 related confinement measures and the rise in uncertainty and mental health issues do not affect the reasons for wishing to have a child and that the reasons for the desire for parenthood do not differ according to one’s sexual identity and did not change since the COVID-19 pandemic. Because the comparison was made with data from a period of more than 10 years, a strong risk of bias in the interpretation of the results must be considered. Therefore, we cannot say that the change in attitude over a 10-year period is attributable to the COVID-19 pandemic with any certainty; indeed other socio-cultural and political events may have (also) played a role.

Fourth, our results revealed that being heterosexual, of a younger age and living in an urban city were significantly positively – and being childless was significantly negatively – associated with the desire to become a parent. The stronger wish for parenthood in heterosexuals is in line with previous studies reporting lower aspirations for parenthood in minoritized sexual identities compared to heterosexuals [49, 50]. However, the present study has only focused on the desire for parenthood. In order to gain a better understanding of the role of family planning in minoritized sexual identities, future research should instead look at parenthood aspiration [51], as it includes not only parenthood desires but also parenthood expectations and parenthood intentions. With regards to being childless as a predictor of desire to become a parent, it is important to note that childlessness should be viewed on a voluntary-involuntary continuum [52]: While some individuals wish to be childless (voluntary), others have a desire to become a parent (involuntary). Hence, future research should include this continuum when examining factors that influence the desires of parenthood. Moreover, a review on studies that have been published between 1999 and 2009 reported that being a parent as a young adult is associated with lower psychological well-being and increased levels of stress mainly due to partnership problems and financial insecurity [53]. This could lead to a decrease in desire for further children. In contrast, a Danish study indicated a correlation between having the first child and greater levels in psychological well-being, whereas having additional children was related to reduced psychological well-being [54]. Still, the actual desire for parenthood was described as a positive predictor for future psychological well-being and a negative predictor for depressive symptoms [55]. Furthermore, results of our study revealed that living in urban areas is associated with the desire for parenthood. This finding is not in line with previous studies which indicated that individuals living in capital cities show lower desire for having children than people living in rural areas [56, 57]. One possible explanation may be the age of participants: In our study, younger age was positively associated with the desire for parenthood. In a representative Australian sample, the desire for parenthood decreased significantly in women aged over 35 years during a COVID-19 related lockdown in 2020. This negative effect was not found in the younger group. The psychological impact of the lockdown during the pandemic with the rise of uncertainty about the future has been shown to be age-dependent: Being older has been found to be associated with greater health concerns and economic anxieties [58, 59] which may explain the decrease in desire for parenthood in older individuals and in parents. Different population demographics in urban and rural areas, including socio-demographic and economic factors such as the higher percentage of singles households in cities and the share of individuals in good economic situations, can affect the desire to become a parent [60, 61]. In contrast to our findings, previous observations show that urban residents realized their plans to become a parent less often than homeowners in rural areas. This supports the relevance of home ownership in rural areas on the wish to become a parent [62, 63].

Concerning the motives for or against the desire for parenthood, higher endorsement of “*Desire for emotional stabilization”* and lower endorsement of “*Fear of personal constraints*” and “*Desire for social recognition*” were significantly correlated with the desire for parenthood. In the latent state-trait personality theory [64], the above-mentioned variables can be viewed as states since they influence the desire for parenthood. These findings are in line with previous research. For example, a study on parenthood desires in relation to sexual orientation and gender identity found that, for both groups, emotional motives had a greater influence on parenting desire than a desire for emotional stabilization or fear of personal constraints. However, in both groups the financial situation was reported as the most important external factor influencing the realization of parenthood desire. With regard to the domain “*Fear of personal constraints”*, another study found that lesbian women report higher social and economic costs involved with parenthood than did heterosexual women, which may explain some of the observed differences in parenthood desires between heterosexual and lesbian women [65].

While this is the first study to examine the desire for parenthood and motives for and against the desire for a child among cis-individuals with minoritized sexual identities compared to cis-heterosexual individuals during the COVID-19 pandemic, literature on the impact of an unfulfilled wish for a child on psychological and physical health is still sparse. A prospective cohort study on women with an unfulfilled wish for a child undergoing assisted reproductive technology treatment showed not only higher levels of stress and depression, but also increased salivary cortisol levels compared to the general female population [66]. Further studies are required to determine the impact of an unfulfilled wish for a child on mental and physical health more intricately.

The current study was conducted with some notable strengths, including the large size of the national sample with a high participation rate of individuals with minoritized sexual identities. The great majority of participants were below 36 years of age. Consequently, the study sample is representative of the most common age range for German individuals to conceive a child (according to the German Federal Statistical Office). On average, German women have a mean age of 30.2 years at the birth of their first child, while the mean paternal age is 33.2 years [67]. To the best of our knowledge, this is the first investigation to compare the desire for parenthood in heterosexuals and individuals with minoritized sexual identities at the time of COVID-19.

Some limitations should be considered when interpreting the study’s findings. First, depressive symptoms were measured by a self-report screening tool (PHQ-4) which cannot provide clinical diagnosis. Self-report questionnaires may show lower rates of identification of mood disorders such as depression as compared with a comprehensive psychological or medical examination [68]. To achieve greater objectivity, further research samples should include participants with clinically-diagnosed psychiatric disorders. Secondly, our study was cross-sectional, with data obtained at one timepoint during the first national COVID-19 lockdown in Germany and therefore no longitudinal analysis was possible. Third, the sampling strategy might have led to sampling bias concerning age and sexual orientation: Participants of the age of 35 years or younger may be considered as overrepresented in both of our participant groups, at least relative to previous studies: Most past research on comparisons with individuals with minoritized sexual identities present data where heterosexual participants are older than the group of individuals with minoritized sexual identities. Consequently, one must be conscious of the probability that the current study population represents a predominance of older individuals with minoritized sexual identities. Future research on the desire for parenthood and reproductive health of individuals with minoritized sexual identities during pandemics should focus on longitudinal research.

## Conclusion

Being part of a minoritized sexual identity community seems to be associated with a higher level of depressive symptoms when compared to heterosexuals. Moreover, our findings show that depressive symptoms and being childless are negatively associated with the desire for parenthood, and that being heterosexual and living in an urban area are positively associated with the desire to become a parent. Our findings underline the unmet need during this pandemic for social, psychological and medical support in regards to family-planning and relating to the desire for parenthood. While this is the first study to examine the desire for parenthood and motives for and against the desire for a child among cis-individuals with minoritized sexual identities compared to cis-heterosexual individuals during the COVID-19 pandemic, further studies are required to determine to what extent an unfulfilled wish for a child affects mental and physical health during global pandemic crises. This will be particularly important in better understanding and supporting vulnerable groups such as individuals with minoritized sexual identities. Access to mental healthcare and resources such as peer support groups within queer communities should be increased during the COVID-19 pandemic. In particular, advanced services such as telemedicine can reduce barriers to making these services readily available during a pandemic. Moreover, close collaboration between primary and mental healthcare systems as well as queer communities should be considered to improve immediate changes in daily routines and implement recommendations in further clinical guidelines.

## Data Availability

Data is stored in a non-publicly available repository. Data is however available from the corresponding author on request.

## Abbreviations

ANOVA: Analysis of Variance
COVID-19: Coronavirus disease
d: Cohen’s d
LKM-20: Leipzig questionnaire on motives for having a child, 20 items
LMU Munich: Ludwig-Maximilians-Universität München
LSD: Fisher’s Least Significant Difference
M: Mean value
p: P-value
PHQ-4: Patient Health Questionnaire, 4 items
SARS-COV 2: Severe acute respiratory syndrome coronavirus type 2
SD: Standard deviation
SPSS: Statistic Package for Social Sciences
