# Publisher Correction: The psychological burden of COVID-19 on the desire for parenthood in minoritized sexual identities: a study on depressive symptoms and family planning in Germany

**DOI:** 10.1186/s12889-023-15385-5

**Published:** 2023-03-30

**Authors:** Falk Batz, Eva Lermer, Sonia Lech, Grace O’Malley, Alaleh Zati zehni, Davina Zenz-Spitzweg, Sven Mahner, Joachim Behr, Christian J. Thaler, Pichit Buspavanich

**Affiliations:** 1 Department of Obstetrics and Gynecology, Center for Gynecological Endocrinology and Reproductive Medicine, University Hospital, LMU Munich, Munich, Germany; 2 Center for Leadership and People Management, LMU Munich, Munich, Germany; 3 Department of Business Psychology, Augsburg University of Applied Sciences, Augsburg, Germany; 4 Institute of Medical Sociology and Rehabilitation Science, Department of Psychiatry and Psychotherapy, Charité – Universitätsmedizin Berlin, corporate member of Freie Universität Berlin and Humboldt- Universität zu Berlin, Berlin, Germany; 5 Department of Paediatric Oncology/Haematology, Charité – Universitätsmedizin Berlin, corporate member of Freie Universität Berlin and Humboldt-Universität zu Berlin, Berlin, Germany; 6 Applied Business and Media Psychology, Ansbach University of Applied Sciences, Ansbach, Germany; 7 Department of Psychiatry, Psychotherapy and Psychosomatics, Brandenburg Medical School Theodor Fontane, Fehrbelliner Str. 38, 16816 Neuruppin, Germany; 8 Faculty of Health Sciences Brandenburg, Joint Faculty of the University of Potsdam, Brandenburg University of Technology Cottbus-Senftenberg and Brandenburg Medical School, Potsdam, Germany; 9 Research Unit Gender in Medicine, Department of Psychiatry and Psychotherapy, Institute of Sexology and Sexual Medicine, Charité – Universitätsmedizin Berlin, corporate member of Freie Universität Berlin and Humboldt-Universität zu Berlin, Berlin, Germany

**BMC Public Health (2023) 23:232.**

10.1186/s12889-023-15127-7.

Due to an error in the publication process the values in Tables 1 and 3 are not properly aligned. The original article has been updated to correct this.

